# Expression of Adenosine A_2B_ Receptor and Adenosine Deaminase in Rabbit Gastric Mucosa ECL Cells

**DOI:** 10.3390/molecules22040625

**Published:** 2017-04-12

**Authors:** Rosa María Arin, Ana Isabel Vallejo, Yuri Rueda, Olatz Fresnedo, Begoña Ochoa

**Affiliations:** Department of Physiology, Faculty of Medicine and Nursing, University of the Basque Country UPV/EHU, Sarriena s/n, 48940 Leioa, Bizkaia, Spain; anaivallejo@gmail.com (A.I.V.); yuri.rueda@ehu.eus (Y.R.); olatz.fresnedo@ehu.eus (O.F.); begona.ochoa@ehu.eus (B.O.)

**Keywords:** adenosine, adenosine deaminase, G protein-coupled receptor, gastric neuroendocrine cell, enterochromaffin-like (ECL) cell

## Abstract

Adenosine is readily available to the glandular epithelium of the stomach. Formed continuously in intracellular and extracellular locations, it is notably produced from ATP released in enteric cotransmission. Adenosine analogs modulate chloride secretion in gastric glands and activate acid secretion in isolated parietal cells through A_2B_ adenosine receptor (A2BR) binding. A functional link between surface A2BR and adenosine deaminase (ADA) was found in parietal cells, but whether this connection is a general feature of gastric mucosa cells is unknown. Here we examine whether A2BR is expressed at the membrane of histamine-producing enterochromaffin-like (ECL) cells, the major endocrine cell type in the oxyntic mucosa, and if so, whether it has a vicinity relationship with ADA. We used a highly homogeneous population of rabbit ECL cells (size 7.5–10 µm) after purification by elutriation centrifugation. The surface expression of A2BR and ADA proteins was assessed by flow cytometry and confocal microscopy. Our findings demonstrate that A2BR and ADA are partially coexpressed at the gastric ECL cell surface and that A2BR is functional, with regard to binding of adenosine analogs and adenylate cyclase activation. The physiological relevance of A2BR and ADA association in regulating histamine release is yet to be explained.

## 1. Introduction

Adenosine (Ado) is a regulatory metabolite that modulates a broad spectrum of processes in many cells of the gastrointestinal tract [1,2]. Ado is continuously produced in intracellular and extracellular locations. In healthy, unstressed tissues, extracellular Ado has a very short-life due to a compensation of concentrations between the extra and the intracellular compartments, which is mainly achieved via equilibrative nucleoside transporters (ENTs) and a rapid metabolism. The Ado basal levels in the interstitial fluid are between 30 and 300 nM [3]. However, under pathophysiological conditions, such as metabolic stress or during inflammation-associated tissue hypoxia or ischemia, Ado generation can exceed the removal capacity resulting in markedly increased extracellular concentrations [4]. Uncontrolled release from cells with damaged plasma membranes—such as that resulting from mechanical stress or trauma—also provides large increases of extracellular purine nucleosides and nucleotides (ATP levels in cells are typically 3–5 mM). In addition, ATP is a cotransmitter [2,5] and this phenomenon is particularly evident in enteric nerves (excellent reviews can be found in references [2,6]). Intrinsic enteric neurons release ATP, which, in addition to serving as a purinergic transmitter [7], can be metabolized to Ado. Extracellular ATP can be ultimately converted into Ado by the sequential action of two families of enzymes: ectonucleoside triphosphate diphosphohydrolase (NTPDase or CD39) which converts ATP to AMP, and ecto-5′-nucleotidase (CD73), which converts AMP to adenosine.

Structurally, Ado receptors are members of the seven transmembrane receptor superfamily and they couple to G proteins [8]. Of the four subtypes of Ado receptors (A_1_, A_2A_, A_2B_ and A_3_), the A_2B_ adenosine receptor (A2BR) still remains the most enigmatic subtype because of the relatively low potency of Ado at this receptor (EC_50_ value of 24 µM) [9], and the very few specific agonists that have been described so far. However, there is a growing interest in A2BR, as it has been found to play a role in several diseases, including cardiovascular disease and cancer [10,11,12,13]. For example, it was found that during inflammatory ischemia the local Ado level is elevated to levels sufficient for A2BR activation [12] and that limited oxygen availability alters Ado signaling at the receptor level, with A2BR being specifically overexpressed by the binding of the central regulator of oxygen homeostasis hypoxia-inducible factor (HIF)-1α to its gene promoter [14,15]. This suggests that A2BR may contribute to dampening inflammation during tissue hypoxia or to fine-tuning other tissue responses when Ado concentration and the density of A2BR is abundant.

Gastric acid secretion is a tightly regulated process. The parietal cell of the gastric mucosa is responsible for the release of hydrochloric acid (HCl) into the gastric lumen. This secretory process is regulated by a highly coordinated interaction with other specialized cells of the stomach, mainly the histamine-secreting enterochromaffin-like (ECL) cells in the body and fundus (the oxyntic area), the gastrin-secreting G cells in the antrum that stimulate acid secretion [16,17], and the D cells in the antrum, body, and fundus of the stomach secreting inhibitory somatostatin [18]. Globally, a collection of neural stimuli (e.g., acetylcholine (ACh)) and endocrine and paracrine agents-acting directly at the parietal cell or indirectly on the regulatory cells mentioned above-, as well as mechanical and chemical stimuli, participate in the physiology of acid secretion (e.g., see reviews [2,17,19]). The primary stimulatory processes are considered to be of histaminergic nature via Gs-coupled H_2_ receptor activation and of cholinergic nature via activation of Gq-coupled muscarinic M_3_ receptors or receptors for gastrin [20], but other actors are also on the stage. For example, we recently uncovered that gastric parietal cells also possess functional A2BR receptors that couple to Gs to stimulate HCL production upon activation [21].

Adenosine deaminase (ADA) is the enzyme that degrades extracellular Ado by catalyzing the irreversible deamination of Ado to inosine. Besides being present in the cytosol and the nucleus, ADA is anchored to the cell surface and determined to be an ectoenzyme [22]. Four anchor proteins are known for ADA: the Ado A_1_, A_2A_ and A_2B_ receptors and CD26 [23,24,25,26]. Surface ADA is a multifunctional protein that, apart from degrading extracellular Ado, can act as a communication bridge between cells and as a co-stimulating molecule in certain activation processes [23,27]. Several in depth studies have considerably improved our understanding of the functional relationship between Ado receptors and ADA [23,24,25,26,27,28,29,30,31]. Interestingly, A2BR was found to mediate ADA docking in CHO cells and Jurkat cells [30], whereas, in dendritic cells, the complex A2BR-ADA was found to promote an immune response triggered by a cell-adhesion co-stimulatory signal [23]. In a previous work, we showed that A2BR is expressed in close apposition to ADA on the cell surface of gastric parietal cells, and that this is especially important in cell-to-cell contact areas [31]. Hence, work in a variety of cell types and cellular settings suggests that ADA may be a more general binding partner of A2BR than anticipated.

The parietal cell-ECL cell axis is the major governor of gastric acid secretion. ECL cells constitute the predominant endocrine cell type in the acid-producing part of the stomach in mammals [32]. They respond to gastrin by releasing histamine and to somatostatin by reducing histamine release [32,33], but the response of the ECL cell to Ado has not been explored. In this context, and using a highly homogeneous population of ECL cells, we investigated whether the histamine-producing ECL cells express functional A2BR in their surface and, if so, whether it colocalizes with ADA. 

## 2. Results

**Figure 1:**
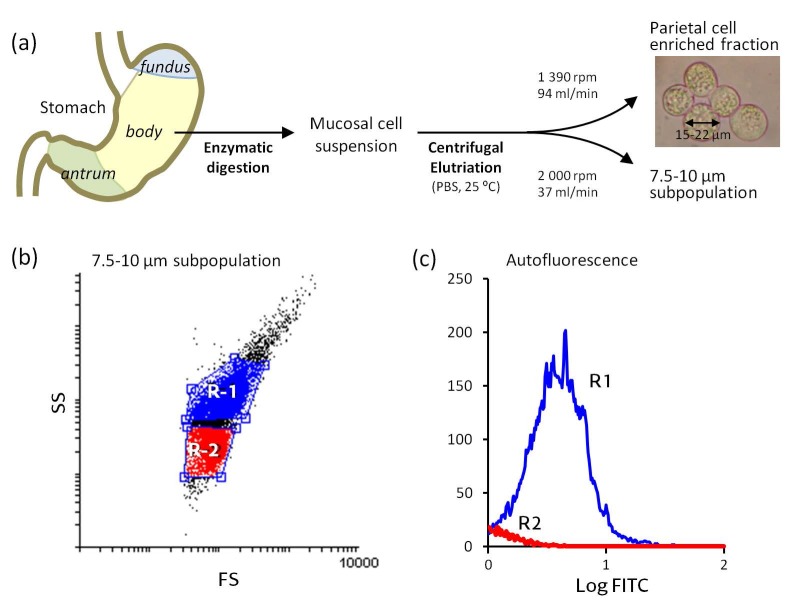
Scheme of the enterochromaffin-like (ECL) cell separation and flow cytometry characterization. (**a**) ECL cells were isolated from the gastric mucosa of the rabbit stomach body region by centrifugal elutriation; particle size distribution was analyzed using a Coulter Counter Multisizer II; (**b**) Spot diagram of the flow cytometry analysis of a representative ECL cell preparation (*n* = 4). The X-axis represents frontal dispersion of light (FS) and the Y-axis represents lateral dispersion (SS); (**c**) Autofluorescence in R1 and R2 cell subpopulations (excitation at 488 nm and emission at 525 nm). The X-axis represents fluorescence intensity (log FITC, arbitrary units) and the Y-axis represents the number of cells that show fluorescence (events). R1 meets the characteristics of ECL cells: size (7.5–10 µm) and elevated cytoplasmic complexity and autofluorescence [33].

ECL cells are predominantly located in the basal half of the acid-producing mucosa. In this work, we isolated ECL cells from the body (corpus) region of the rabbit stomach by enzymatic digestion of the mucosa followed by separation of this specific cell population from other mucosal cells using centrifugal elutriation [34] (Figure 1a). ECL cells were used without any prior stimulation, and their size, cytoplasmic complexity and autofluorescence were characterized. The ECL cell is a small, dense cell (8–10 µm) [35] that contains abundant granules and secretory vesicles storing histamine and presents high cytoplasmic complexity [32,33,35]. Autofluorescence is a hallmark of the ECL cell due to the presence of histamine in the secretory vesicles [36]. The particle size distribution analysis, performed with Coulter Counter equipment, yielded that cells in our ECL populations consistently had a diameter between 7.5 and 10 µm. As shown in the dot plot of the flow cytometry, two cellular subpopulations can be differentiated in our preparations (Figure 1b). The X-axis represents frontal dispersion of light (FS), which indicates cell size, and the Y-axis represents lateral dispersion (SS), indicative of cell complexity. In Figure 1c, cell autofluorescence distribution is shown. Hence, the R1 subpopulation meets the criteria of ECL cells: size (7.5–10 µm), cytoplasmic complexity distribution and autofluorescence. Because D cells and ECL cells have similar size and shape characteristics and D cells lack autofluorescence [36], the R2 subpopulation may also contain contaminant D cells. The R1 subpopulation is predominant and accounts for more than 70% of the cells that were submitted to immunostaining. It is important to mention that, to date, there are some reports supporting A_1_ [37] and A_2A_ [38] localization in D cells, but there is no such evidence for A_3_ and A2BR [39].

**Figure 2:**
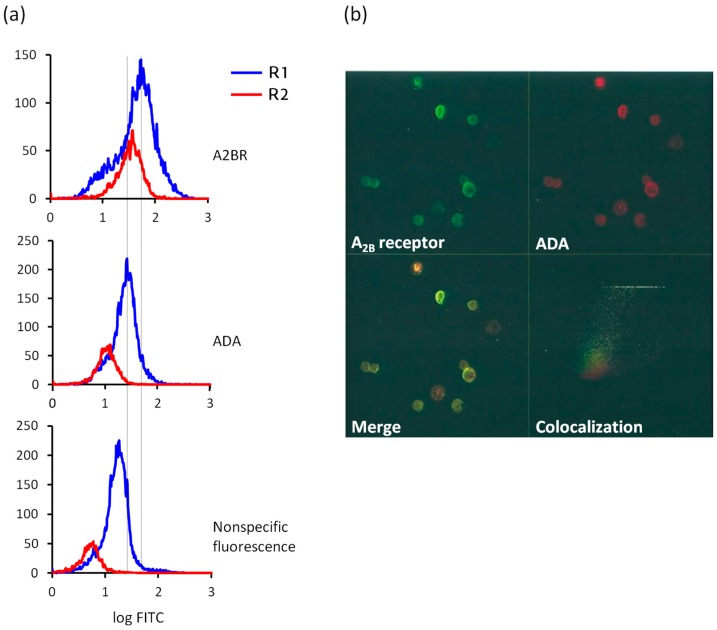
Immunodetection of cell surface adenosine deaminase (ADA) and A2BR in ECL cells by flow cytometry and confocal microscopy. (**a**) Monoparametric representation of fluorescence in R1 and R2 cell subpopulations. The minimum number of cells used was 20,000. The X-axis represents FITC fluorescence intensity (log FITC; arbitrary units) and the Y-axis represents the number of cells that show fluorescence (events). Top bottom: anti-A2BR-FITC fluorescence, anti-ADA-FITC fluorescence and nonspecific fluorescence. Histograms were analyzed with flowing software; (**b**) For confocal microscopy, cells were fixed and labeled with anti-A2BR-FITC (upper left image, green) and anti-ADA-TRITC (upper right image, red). The merge of the two images shows partial colocalization of cell surface A2BR and ADA (lower left image, yellow). The lower right image shows the confocal cytofluorogram in which yellow represents colocalization of the two proteins.

With the aim of exploring whether gastric ECL cells expressed A2BR and ADA on their surface, we performed immunostaining experiments using flow cytometry (Figure 2a) and confocal microscopy (Figure 2b). In a previous study, we confirmed by western blotting experiments that the antibodies here used, that had been raised against the human proteins [28,30,40,41], recognized monospecifically A2BR and ADA in rabbit tissue [31]. 

Flow cytometry analysis revealed the distribution of ADA and A2BR in immunostained R1 and R2 cell subpopulations (Figure 2a). Cytometry was also performed using only the secondary antibody in order to assess the fluorescence intensity due to nonspecific binding; it accounts for 31% and 14% of the labeling for A2BR in R1 and R2 cells, and for 71% and 49% of the labeling for ADA in R1 and R2 cells, respectively. Those data were used to calculate the number of cells (%) that expressed A2BR or ADA with a fluorescence intensity higher than the nonspecific, basal fluorescence, which was 63% and 92% of A2BR-positive R1 and R2 cells, and 32% and 67% of ADA-positive R1 cells of R2 cells. 

The colocalization of A2BR and ADA at the ECL cell plasma membrane was studied by confocal microscopy of nonpermeabilized cell preparations (Figure 2b). ECL cells showed significant surface expression of A2BR (upper left image, green) and ADA (upper right image, red). The merge of both signals (lower left image) indicates that some ECL cells presented immunoreactivity only to A2BR (green) and others only to ADA (red), while others coexpressed the two antigens (yellow). The cytofluorogram in the fourth panel in Figure 2b shows that the colocalizing areas correspond to the pixels with the highest fluorescence intensity, indicating highly specific vicinity of A2BR and ADA, though there is a moderate degree of A2BR and ADA colocalization in ECL cells.

**Figure 3:**
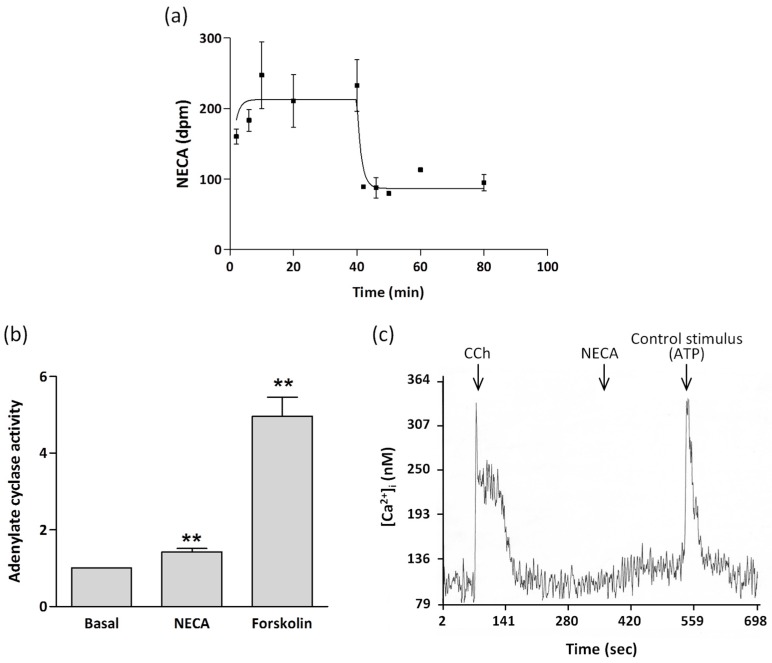
Association-dissociation of [^3^H]NECA to ECL cell membranes from rabbit gastric mucosa, and NECA-promoted effects in adenylate cyclase activity and Ca^2+^ levels. (**a**) After membranes (0.5 mg protein/mL) were incubated with 50 nM [^3^H]NECA for 40 min, dissociation was provoked by 50 µM NECA. Values are the mean ± SEM of *n* = 3. Where absent, the error bar lies within the symbol; (**b**) Adenylate cyclase activity was determined in cell membranes in the absence (basal) or presence of 10^−4^ M NECA or 10^−4^ M forskolin. Data were normalized with the basal value, that averaged 6.3 ± 2.8 pmol of cAMP produced/mg protein. Values are the mean ± SEM of *n* = 3. ** *p* < 0.01; (**c**) A single ECL cell was sequentially challenged with 20 µM carbachol (CCh) and 10 µM NECA. ATP (50 µM) acts as a positive control. Cytosolic calcium was measured by microfluorimetry, *n* > 15.

A2BR is a low-affinity Ado receptor [8,9]. To investigate the functional significance of A2BR in ECL cells, we analyzed the kinetic behavior of membranes purified by ultracentrifugation from ECL cells towards [^3^H]5′-*N*-ethyl-carboxamido-adenosine (NECA). As shown in Figure 3a, the association of 50 nM [^3^H]NECA to membranes revealed that equilibrium was rapidly reached. Figure 3a also shows the efficient displacement of radiolabeling by 50 µM unlabeled NECA. The association/dissociation experimental data were compared with models of one or two binding site populations using GraphPad software. The data best fitted a one site equation model (R^2^ = 0.88), giving a Kd of 103.8 ± 0.3 µM. NECA is a nonspecific agonist for Ado receptors. However, the fact that data fit to a unique binding site and that the obtained Kd value is in the range of those described for A2BR in many experimental models [8,21,41,42,43,44], strongly suggests that A2BR is mediating NECA binding; it is several orders of magnitude above those reported for the high-affinity receptors A_1_ and A_2A_ and also for the so called low-affinity A_3_ Ado receptor [9,43,44].

A2BR is known to couple to Gs and Gq proteins [42,45]. Most A_2B_ Ado receptors are coupled to Gs proteins and activate adenylate cyclase, resulting in the intracellular production of cAMP and subsequent activation of PKA. However, activation of calcium-dependent mechanisms involving the Gq family of proteins to activate PLC and increase intracellular Ca^2+^ has been reported in some cell types [11,45]. To determine the signal transduction system operating in ECL cells, we evaluated adenylate cyclase act**i**vity and mobilization of intracellular Ca^2+^ in response to A2BR activation.

We confirmed that A2BR activation was coupled to Gs stimulation by direct measurement of the cAMP-generating capacity of membranes isolated by ultracentrifugation from ECL cells (Figure 3b). Control experiments in which membranes were treated with 10^−4^ M forskolin, a direct activator of adenylate cyclase, resulted in high increases of cAMP production. In the same way, 10^−4^ M NECA led to a moderate, but consistent and significant increase of about 45% in adenylate cyclase activity. Similarly, 10^−4^ M 2-chloro-adenosine (2-CADO) also increased cAMP production (data not shown).

To test whether transduction by Gq was also involved in A2BR activation, cytosolic calcium waves were recorded by microfluorimetry in individual ECL cells in response to various effectors (Figure 3c). We observed the elevation of intracellular Ca^2+^ produced by carbachol (CCh), a muscarinic agonist that has been reported to increase intracellular Ca^2+^ in ECL cells [46], manifested as an initial spike, representing Ca^2+^ release from intracellular stores, followed by a lower level plateau caused by an inward current of Ca^2+^. In contrast, no calcium mobilization occurred after challenging ECL cells with NECA. As expected [47,48], not all the tested cells responded to CCh.

Collectively, our findings indicate that rabbit gastric ECL cells express A2BR and ADA at the cell surface and that A2BR couples to Gs proteins, and not to Gi or Gq, upon agonist binding.

## 3. Discussion

The ECL cell of the oxyntic mucosa plays a critical role in the regulation of acid secretion through the production and secretion of histamine. ECL cells respond to gastrin by releasing histamine and to somatostatin by inhibiting histamine release. In this study, we have applied an elutriation centrifugation procedure to obtain a cell suspension enriched in ECL cells from the body area of the rabbit stomach mucosa. These cells are representative populations of native (nontransformed) primary ECL cells at rest; therefore, it is possible to identify the surface receptors to which these cells can putatively respond. We used specific antibodies in flow cytometry and confocal microscopy experiments to assess the expression of A2BR, a receptor for Ado operating mainly via adenylate cyclase activation, and ADA, the enzyme that is capable of inactivating endogenous Ado on the cell surface and of binding Ado receptors, affecting their functionality [24,25]. We show for the first time that ECL cells express the Gs-coupled A2BR as well as ADA on their surface.

The regulatory role of Ado in gastric acid secretion is inconclusive. Broadly speaking, it seems evident that Ado has species-dependent actions on regulating acid secretion, which may be inhibitory or stimulatory, and direct or indirect. Controversy mainly derives from the complexity of the network that controls acid release, which makes it challenging to dissect the role of each individual component (for a recent review, see Ref. [2]), and from the fact that Ado is always present within and outside cells. This is exemplified in the perfused stomach of the A_2A_ knockout mice, by showing that Ado has dual actions on regulating the release of inhibitory somatostatin: stimulatory at high concentrations through A_2A_ receptor and inhibitory at low concentrations via A_1_ receptor activation, with no role for A_2B_ and A_3_ [39]. In previous pharmacological studies we demonstrated that Ado analogs stimulate HCl secretion by acting directly at the parietal cell through Gs-coupled A2BR binding [21]. Also in rabbit gastric mucosa, we show here that most ECL cells express A2BR and some of them coexpress ADA, and that A2BR activation by the Ado analog NECA triggers adenylate cyclase activation, which might affect the ECL cell function. 

**Figure 4:**
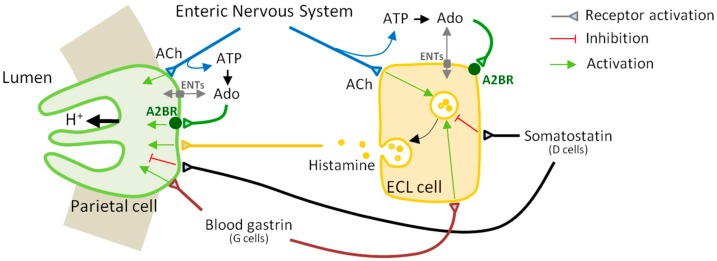
A parietal cell-ECL cell axis governs gastric acid secretion. The main stimulants of acid secretion are histamine, gastrin and acetylcholine (ACh) and the main inhibitor is somatostatin. Adenosine (Ado) also stimulates acid secretion by direct binding to adenosine A_2B_ receptor (A2BR) in the parietal cell. A2BR and ecto-adenosine deaminase (ADA) are also expressed on the histamine-secreting ECL cell surface. Extracellular levels of Ado result from the hydrolysis of its precursor nucleotides, including the ATP released from cells and intramural neurons of the stomach, besides the activities of equilibrative nucleoside transporters (ENTs) and ecto-adenosine deaminase.

The main hormonal and paracrine stimulant of acid secretion is histamine released from ECL cells, and the main hormonal stimulant of histamine release is gastrin secreted from G cells (Figure 4). Gastrin acts as a ligand for the CCK-2 receptor on ECL cells, which activates PLC to induce a rise in cytosolic Ca^2+^ resulting in exocytosis and the release of histamine [49]. Histamine release is evident within 5 min after gastrin stimulation and the Ca^2+^ signal is virtually instantaneous [50]. Stimulation of ECL cells by acetylcholine or the neuropeptide PACAP (pituitary adenylate cyclase-activating polypeptide), a member of the vasoactive intestinal peptide family expressed in the enteric nerves, also results in the release of histamine and is accompanied by a change in Ca^2+^ concentration [50]. Variations in cAMP levels do not seem to affect this secretion process; consequently, it seems unlikely that A2BR activation can have a direct effect on exocytosis and the release of histamine from the secretory organelles in ECL cells. It was only in the rat that histamine secretion from ECL cells was found to be activated by epinephrine and forskolin [48].

Histamine is generated through the action of l-histidine decarboxylase (HDC) [51]. The best characterized stimuli of HDC gene expression and activity in the ECL cell are gastrin, via CCK-2 receptor binding and PLC activation [47], and PACAP, involving binding to PACAP receptor PAC1 [52] and both the adenylate cyclase/PKA and the PLC/PKC signaling pathways [53]. Activation of adenylate cyclase by forskolin (10^−4^ M) was found to markedly activate human HDC promoter activity in the PAC1-expressing neuroendocrine cell line PC12, stably transfected with the gastrin/CCK-2 receptor [53]. Therefore, it is possible that pathophysiological stimuli other than PACAP induce cAMP-mediated histamine generation in ECL cells, which agrees with a possible A2BR-mediated role for Ado in histamine synthesis in those conditions—such as inflammatory hypoxia [12]—in which interstitial adenosine concentrations rise.

It is pertinent to point out that a contribution of D cells to the findings reported in this study cannot be ruled out. Certainly, the R2 fraction, representing a low proportion of the total cell preparations analyzed, may be contaminated with somatostatin-secreting D cells from the body of the stomach. As mentioned before, ECL and D cells are of similar size and shape but D cells lack autofluorescence [36]. Autofluorescence is due to the histamine stored in secretory granules of the ECL cells. Even though ECL cells were not stimulated with any secretagogue prior to flow cytometry, and shear forces in centrifugal elutriation were mild (viability was 96%), a small subset of primary ECL cells may have activated, displaying low fluorescence because of the emptying of the secretory vesicles [36]. Hence, the possibility exists that R2 fraction contains a mixture of histamine-depleted ECL cells and D cells. No evidence exists of A2BR expression in D cells [39], though there are reports supporting the presence of A_1_ [37] and A_2A_ [38]. What is remarkable in the current study is that most ECL cells (R1 cells) are positive to A2BR and about half of them express ADA on their surface, indicating that surface expression of A2BR is nearly generalized on nonstimulated, resting ECL cells and higher than ADA expression. 

In conclusion, we have identified A2BR as an Ado receptor that partially interacts with ADA at the plasma membrane of ECL cells originating from the body of the rabbit stomach and that A2BR couples to Gs upon activation. These findings raise the question as to whether Ado itself has an impact on the physiology of histamine secretion and what the functional relevance of A2BR in ECL cells is. It is conceivable that Ado might induce HDC promoter activity and histamine synthesis by acting via A2BR and adenylate cyclase activation, in a similar fashion as PACAP. This is an important consideration for future studies given the elevated local adenosine conce that are presumably found during mucosal inflammation, which may hit histamine production in ECL cells and contribute to modulate acid secretion in an effect complementary to that caused directly on the acid-secreting parietal cell.

## 4. Materials and Methods

### 4.1. Materials

[^3^H]5′-*N*-ethyl-carboxamido-adenosine (NECA) and [^3^H]cAMP were obtained from GE Healthcare Life Sciences (Little Chalfont, UK), and ATP, NECA and CCh were obtained from Sigma-Aldrich Química S.L. (Madrid, Spain).

### 4.2. Isolation and Enrichment of ECL Cells

Male and female New Zealand rabbits (2.5–4 kg bw) were used in accordance with the Spanish (RD 1201/2005) and European (2003/65/CE Directive and 2007/526/CE Recommendation) guidelines for the use of laboratory animals. Rabbits were housed in climate-controlled specific pathogen-free facilities with a 12 h light/dark cycle, with free access to standard food and water. For each experiment, two rabbits were used. The isolation of gastric mucosa cells by pronase and collagenase digestion of the mucosa was carried out as described in previous works [21,31,54]. Briefly, after perfusion with PBS, the stomach was removed and the fundic and antral regions were discarded. The corpus was rinsed in PBS, the mucosa was separated and minced and the fragments were digested with pronase and type I collagenase in oxygenated (95% O_2_ and 5% CO_2_) medium at 37 °C.

Cell separation was performed by counterflow elutriation at room temperature (~25 °C) in a Beckman J-6M/E centrifuge equipped with a JE-6B rotor using a PBS buffer containing 2 mg/mL glucose following the Sanders and Soll protocol [34] with minor modifications. The ECL cell fraction was collected (500 mL) (at 2000 rpm and 37 mL/min) and washed (2400 rpm, 10 min). Size distribution of harvested cells was analyzed in a Coulter-Counter Multisizer II (Hialeah, FL, USA). It was found that 68.4% of cells in the final suspension had a size of 7.5–10 µm. Cell viability, as measured by the Trypan blue exclusion test, averaged 96%.

### 4.3. Immunostaining Experiments: Flow Cytometry and Confocal Microscopy

The staining procedure was conducted exactly as described earlier [31] using anti-human A2BR [28] and anti-human ADA [40]. Nonpermeabilized ECL cells were fixed in 2% (*w*/*v*) paraformaldehyde, 60 mM sucrose in PBS, pH 7.4, for 15 min at room temperature. After washing with 20 mM glycine in PBS, cells were blocked with 1% BSA and incubated with anti-A2BR or anti-ADA antibodies and a FITC-conjugated secondary antibody (Boehringer Mannheim, Barcelona, Spain). Primary antibodies were provided by Prof. R. Franco (University of Barcelona, Barcelona, Spain).

Flow cytometry was performed with an EPICS Profile flow cytometer (Coulter Corporation, Miami, FL, USA). The parameters used to select cell subpopulations for analysis were forward and side light scattering. Cell sorting was performed using the EPICS Profile equipment and data were analyzed using Flowing software (version 2.5.1; Turku Centre for Biotechnology, Turku, Finland).

For confocal microscopy analysis, cells were immunostained as with flow cytometry except that a TRICT-conjugated secondary antibody (Boehringer Mannheim, Barcelona, Spain) was used to visualize ADA. Cells were mounted with Immuno-Fluore mounting medium and scanned using a Leica TCS 4D confocal scanning laser microscope adapted to an inverted Leitz DMIRBE microscope (Leica Lasertechnik GmbH, Heidelberg, Germany). The colocalization analysis was performed by means of Multi Color software (version 2.0; Leica Lasertechnik GmbH).

### 4.4. Isolation of ECL Cell Membranes

Plasma membranes were prepared as described previously [54]. ECL cells (60 × 10^6^ cells/mL) were homogenized with a Potter-Elvehjem homogenizer in ice-cold 20 mM Tris-HCl buffer, pH 7.4, containing 250 mM sucrose, 0.5 mM EDTA, 0.54 mM dithiothreitol, 5 µg/mL leupeptin and 15.7 µg/mL benzamidine. The 700× *g* pellet was discarded and the supernatant was collected and placed on top of a 47% sucrose solution and centrifuged (100,000× *g*, 4 °C, 45 min) in a swinging rotor. Plasma membranes were then collected from the interphase and resuspended in 50 mM Tris-HCl buffer, pH 7.4 (buffer A) and washed (120,000× *g*, 4 °C, 20 min). Membranes were finally resuspended in buffer A and stored at –80 °C until used. The protein concentration of the membranes was measured using the method of Bradford [55].

### 4.5. Radioligand Binding Experiments

Binding assays were carried out as described by Casadó and co-workers [56]. Association experiments were performed by incubating 50 nM [^3^H]NECA (850 nCi/nmol) with 0.5 mg membrane protein/mL in buffer A at room temperature. Dissociation was induced by addding unlabeled NECA after an association period of 40 min. At the times specified, free and membrane-bound radioligands were quickly separated by vacuum filtration through Whatman GF/B filters previously soaked in 0.3% polyethylenimine, pH 10, for 2 h. The filters were sequentially bathed in 6 mL of buffer A and placed in 10 mL of a scintillation cocktail for radioactivity measurement. The experiment was repeated three times and the assays were performed in duplicate.

### 4.6. Determination of Membrane Adenylate Cyclase Activity

Adenylate cyclase activity was determined in cell membranes by quantifying the cAMP generated from ATP using a competitive protein binding procedure [57], as described previously [21]. In brief, after membranes (0.8 mg protein/mL) were preincubated (5 min at 30 °C) in buffer A supplemented with 5 mM Mg_2_Cl and 1 mM DTT, the reaction was initiated by adding 200 μM ATP and was stopped 10 min later. The samples were then centrifuged at 12,000 *g* for 2 min, and 50 μL supernatants were taken for cAMP determination. To each well of a 96-well microplate, 50 μL of [^3^H]cAMP (54 Ci/mmol) and 100 μL of 0.3 mg/mL bovine adrenal PKA were added in a total volume of 250 μL. A standard curve of cAMP was included in each plate. Plates were incubated at 4 °C for 150 min and then freed and PKA-bound [^3^H]cAMP were separated by vacuum filtration through Whatman GF/B filters using a Skatron Micro 96 Harvester. Filters were washed twice with 3 mL of buffer A and placed afterwards in 3 mL of a scintillation cocktail for radioactivity measurement. The experiment was repeated three times and the assays were performed in quadruplicate.

### 4.7. Assessment of Calcium Mobilization in Individual Cells by Microfluorimetry

The cytosolic Ca^2+^ concentration was recorded in single cells using a multiple excitation microfluorescence system (Cairn Research Ltd., Faversham, UK), as described in a previous work [21]. After three washes in medium DMEM:F12 (1:1, *v/v*), cells were seeded (1 × 10^6^–2 × 10^6^ cells) on Matrigel-precoated coverslips and cultured for 1 h at 37 °C in 5% CO_2_ atmosphere in DMEM:F12 (1:1, *v/v*) supplemented with 15 mM HEPES, pH 7.4, 2 mM l-glutamine, 10 nM hydrocortisone, 1 mg/mL insulin, 0.1 mg/mL gentamicin, 0.5 μg/mL transferrin, 5 μg/mL sodium selenite, 10 mM glucose, 5 μg/mL geneticin and 2 mg/mL BSA. Then, cells were washed and loaded with 5 μM fura-2/AM for 60 min at 37 °C. Coverslips were placed in a superperfusion chamber of a Nikon Diaphot microscope thermostatically controlled at 30 °C and the cells were excited alternatively at 340, 360 and 380 nm to monitor the fluorescence emitted at 510 nm. Control and calibration procedures as well as the algorithms used to calculate the cytosolic Ca^2+^ concentration are described previously [58].

### 4.8. Statistical Analyses

All values are given as the mean ± SEM of 3–4 independent experiments. Differences between two groups were assessed by unpaired Student’s two-tailed *t*-test using GraphPad Prism (version 5.02, GraphPad software, La Jolla, CA, USA). Differences were considered significant at a value of *p* ≤ 0.05.
